# Quorum quenching by Est816: a novel approach to control *Porphyromonas gingivalis* pathogenicity

**DOI:** 10.1186/s12903-025-06563-5

**Published:** 2025-07-16

**Authors:** Zelda Ziyi Zhao, Lifeng Guo, Xiangyang Li, Tianfan Cheng, Chun Hung Chu, Jing Zhang

**Affiliations:** 1 Faculty of Dentistry, The University of Hong Kong, Hong Kong SAR, China; 2 College & Hospital of Stomatology, Key Lab. of Oral Diseases Research of Anhui Province, Anhui Medical University, Hefei, 230032 China

**Keywords:** *N*-acyl Homoserine lactones, Quorum sensing, *Porphyromonas gingivalis*, Peri-implantitis, Biofilm

## Abstract

**Background:**

*Porphyromonas gingivalis* (*P. gingivalis*), a keystone pathogen in peri-implantitis, employs quorum sensing (QS) via *N*-acyl homoserine lactones (AHLs) to regulate biofilm formation and virulence. Quorum-quenching enzymes, such as the AHL-lactonase Est816, offer a promising therapeutic strategy to disrupt microbial pathogenicity. This study investigated the anti-biofilm, anti-virulence, immunomodulatory, biocompatibility, and osteogenic properties of Est816 against *P. gingivalis*, exploring its therapeutic potential for peri-implantitis management.

**Methods:**

*P. gingivalis* (ATCC 33277) was cultured on titanium discs and treated with Est816 (*P. gingivalis* + Est816). Biofilm morphology, biomass, viability, and kinetics were assessed using scanning electron microscopy (SEM), crystal violet staining, confocal laser scanning microscope (CLSM), and colony-forming unit (CFU) counting. Exopolysaccharide (EPS) production was quantified via phenol-sulfuric acid assay, while virulence gene expression was analyzed by RT-PCR. Cytotoxicity of Est816 on human oral keratinocytes (HOKs) was assessed using immunofluorescent microscopy. The immunodulatory impact of Est816 on *P. gingivalis* infected human periodontal ligament stem cells (PDLSCs) was assessed via ELISA and RT-PCR. Osteogenic differentiation of PDLSCs was examined by alizarin red staining.

**Results:**

Est816 treatment disrupted biofilm architecture (SEM), reducing biomass (crystal violet: 88% decrease, *p* < 0.001), viability (CLSM: live/dead ratio 0.3 vs. 5.7 control, *p* < 0.05), and CFU counts (2.8-log reduction, *p* < 0.001). EPS production decreased by 44% (*p* < 0.01), and virulence gene expression was significantly suppressed (*rgpA*: 80%, *kgp*: 76%, *fimA*: 73%, *p* < 0.01). Est816 exhibited no cytotoxicity toward HOKs and attenuated pro-inflammatory cytokine secretion in PDLSCs (TNF-α: 2.4-fold, IL-1β: 2.3-fold, IL-6: 11-fold, IL-8: 14-fold, reduction, *p* < 0.05). Furthermore, Est816 alone had no effect on the osteogenic differentiation of PDLSCs; however, it abolished the inhibitory effect of AHLs, significantly enhancing mineralized nodule formation by 1.4-fold (*p* < 0.001) compared to the AHL-treated control.

**Conclusion:**

Est816 exhibited anti-biofilm property, attenuated virulence release in *P. gingivalis*, and counteracted AHL-mediated suppression of osteoblast differentiation in PDLSCs, highlighting its dual therapeutic role in both pathogen inhibition and host tissue regeneration for peri-implantitis.

## Background

*Porphyromonas gingivalis* (*P. gingivalis*), a gram-negative anaerobic bacterium, is a keystone pathogen in the etiology of periodontal diseases, including chronic periodontitis and peri-implantitis. These conditions are characterized by inflammatory destruction of tooth-supporting tissues (periodontitis) or peri-implant bone and soft tissues (peri-implantitis), ultimately leading to tooth loss or implant failure if left untreated [1, 2]. A hallmark of *P. gingivalis* pathogenicity lies in its ability to form resilient biofilms on both natural and artificial surfaces. These biofilms serve as reservoirs for bacterial persistence, enabling evasion of host immune defenses and fostering dysbiosis within the oral microbiome. Critically, biofilm-associated infections are challenging to conventional therapies due to the protective extracellular matrix, which hinders antimicrobial penetration and facilitates horizontal gene transfer, accelerating the development of drug resistance [3]. While mechanical debridement and systemic antibiotics remain first-line treatments, their efficacy is often transient, failing to prevent biofilm reformation or address the underlying virulence mechanisms of *P. gingivalis* [4]. This therapeutic gap underscores the urgent need for strategies that target biofilm resilience while mitigating bacterial pathogenicity and promoting tissue regeneration.

Central to *P. gingivalis* biofilm regulation is quorum sensing, a cell-density-dependent signaling system that coordinates bacterial gene expression and biofilm maturation. In gram-negative bacteria, quorum sensing predominantly relies on *N*-acyl homoserine lactones (AHLs), small diffusible molecules that activate transcriptional regulators upon reaching a threshold concentration. AHLs, initially considered an intra-species signaling molecule, have now been found to play a significant role in inter-species communication as well. In oral polymicrobial biofilms consisting of *Streptococcus oralis*, *Veillonella parvula*, *Fusobacterium nucleatum*, *Aggregatibacter actinomycetemcomitans* (*A. actinomycetemcomitans*), the presence of AHLs enhanced the recruitment of *P. gingivalis*, allowing it to take advantage of the protective and nutrient-rich environment afforded by biofilm formation [5]. Disrupting of AHL-mediated signaling-termed quorum quenching, presents a promising approach to attenuate biofilm formation without inducing bactericidal pressure, thereby minimizing resistance development. Several studies have reported two primary quorum quenching strategies, (i) AHL analogs that competitively inhibit signal reception and (ii) enzymatic degradation of AHLs via lactonases or acylases [6, 7]. Notably, AHL-lactonases, which hydrolyze the lactone ring of AHLs, offer broad-spectrum activity against diverse AHL- variants, making them particularly attractive for targeting polymicrobial infections [5, 6].

Est816, a novel thermostable AHL-lactonase, has demonstrated exceptional catalytic efficiency against all types of medium- and long-chain AHLs, including those produce by periodontal pathogens [8]. Our prior research revealed its capacity to disrupt biofilm formation in pathogenic *A. actinomycetemcomitans*, reducing extracellular polysaccharide synthesis and virulence factor expression [9]. Furthermore, combining Est816 with subinhibitory doses of antibiotics synergistically enhanced effect biofilm eradication, highlighting its potential as an adjuvant therapy for periodontitis [10]. Given the shared reliance on quorum sensing among periodontal pathogens, we hypothesize that Est816 may similarly impair *P. gingivalis* pathogenicity. However, the enzyme’s effects on *P. gingivalis*-specific virulence traits, host-cell interactions, and tissue regenerative processes remain unexplored.

**Fig. 1:**
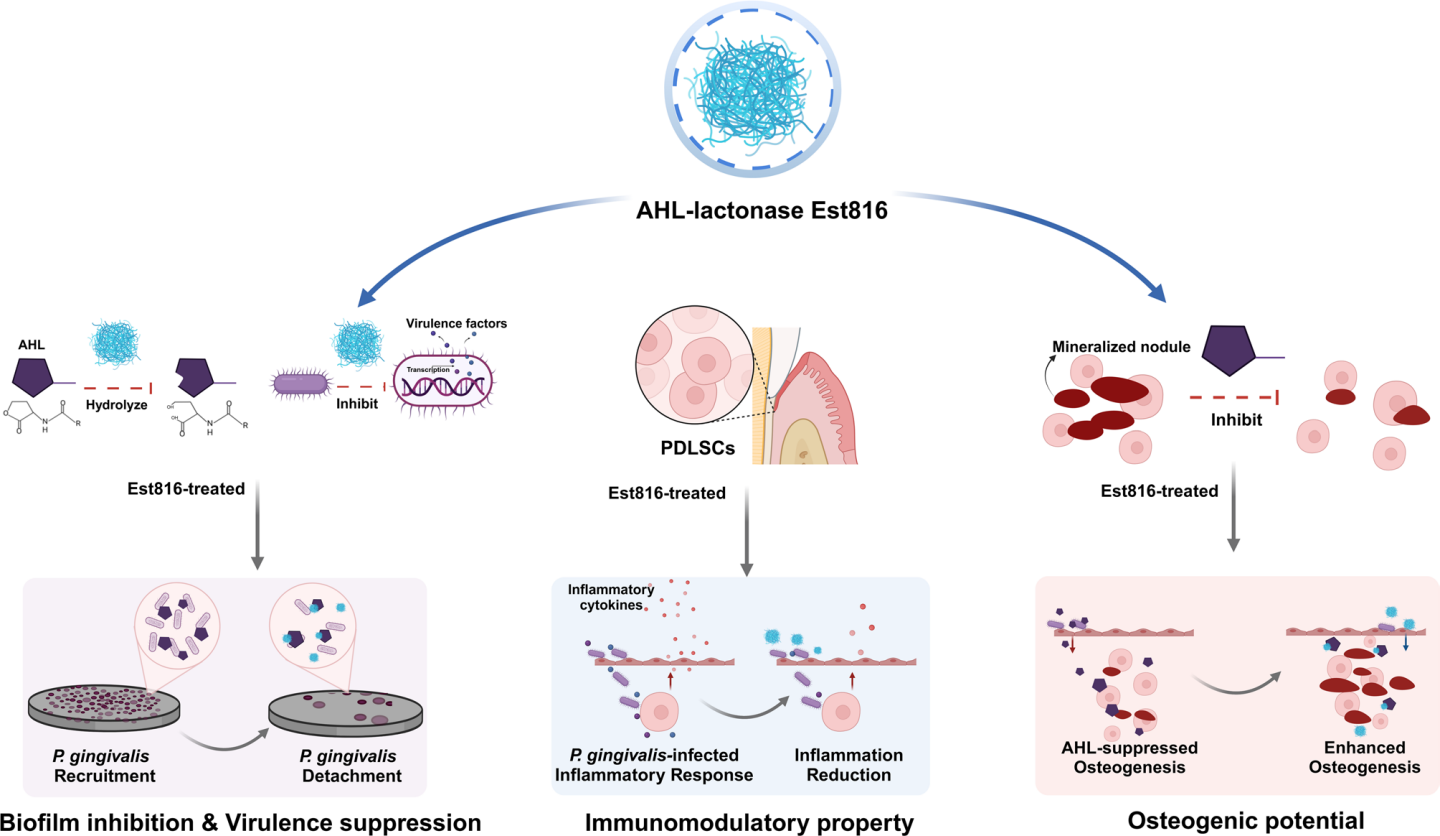
Schematic illustration of the mechanism of Est816 on biofilm inhibition, virulence suppression, immunomodulatory property and osteogenic potential

This study investigates the therapeutic potential of Est816 against *P. gingivalis* through a multidisciplinary approach. We evaluate (i) its anti-biofilm and anti-virulence abilities, (ii) biocompatibility with human oral keratinocytes (HOKs), (iii) immunomodulatory effects on inflammatory cytokine production, and (iv) influence on osteogenic differentiation of human periodontal ligament stem cells (PDLSCs) with or without AHLs (Fig. 1). This study proposes that Est816 not only offers a robust anti-biofilm effect but also supports oral tissue health through immunomodulation and osteogenesis, paving the way for more effective management for peri-implantitis.

## Materials and methods

### Materials

Dimethyl sulfoxide (DMSO), crystal violet solution (1 wt% solution), glutaraldehyde was obtained from Sigma-Aldrich (MO, USA). LIVE/DEAD BacLight (SYTO 9/propidium iodide) Kit was purchased from Invitrogen detection technologies (MA, USA). Fast SYBR Green Master Mix and High-Capacity cDNA Reverse Transcription Kit were ordered from Thermo Fisher Scientific Inc (MA, USA). Total RNA Isolation System Kit was ordered from Promega Corporation (Wisconsin, USA). ELISA Kits (IL-β, TNF-α) were purchased from ProteinTech Group (Chicago, USA). Titanium (Ti) discs (13 mm in diameter and 1 mm in thickness) were purchased from BaojiShengdaxing Corporation (Shanxi, China), and were polished step-by-step using metallographic sandpaper (from 240 # to 2,000 #) to achieve a mirror effect, sonicated with acetone, ethyl alcohol, and deionized water, then dried and ultraviolet disinfected for utilization. The preparation and purification instructions on Est816 were consistent with the previous research, according to which, the molecular mass of Est816 was determined by sodium dodecyl sulfate-polyacrylamide gel electrophoresis (SDS-PAGE) as well [9]. The enzyme activity Est816 was defined as the amount of enzyme required to produce 1 µmol of *p*-nitrophenol using *p*-nitrophenyl acetate as the substrate per minute at 36 °C [8]. For sterilization, Est816 was filtered through 0.22 μm pore-size sterilizing filters.

### Bacteria culture conditions

*P. gingivalis* ATCC 33,277 was obtained from ATCC (VA, USA). The mid-log phase culture of *P. gingivalis* (0.5 McFarland) in supplemented tryptic soy broth (sTSB) [30 g L^− 1^ tryptic soy broth (Difco), 5.0 g L^− 1^ yeast extract (Difco),5.0 µg mL^− 1^ hemin, and 1.0 µg mL^− 1^ vitamin K_1_] was used in subsequent experiments.

### Crystal Violet assay

**Fig. 2:**
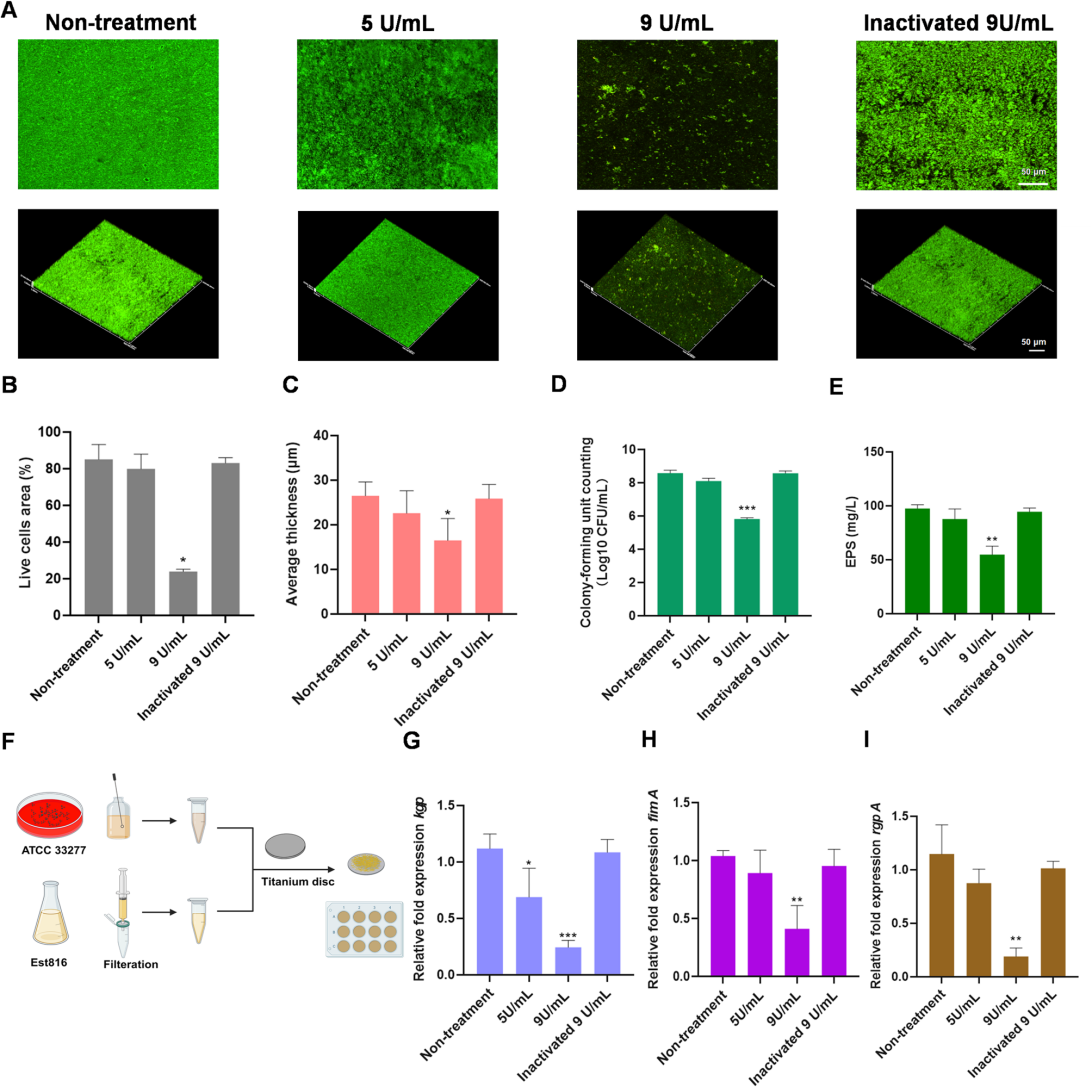
(**A**) CLSM images of *P. gingivalis* biofilm treated with enzymatically activated (5 and 9 U mL^− 1^) and hyperthermia-inactivated Est816 (9 U mL^− 1^) on the surface of glass slips (magnification factor of 20 x). (**B**) Proportion of live cells area quantified from the CLSM images using Image-pro Plus software. (**C**) Average thickness value quantified from the CLSM images using Image-pro Plus software. (**D**) CFU counting of live bacteria in *P. gingivalis* biofilm treated with enzymatically activated (5 and 9 U mL^− 1^) and hyperthermia-inactivated Est816 (9 U mL^− 1^) on Ti discs. (**E**) Exopolysaccharide production quantity from *P. gingivalis* treated with Est816 enzymatically activated (5 and 9 U mL^− 1^) and hyperthermia-inactivated Est816 (9 U mL^− 1^). (**F**) Procedure for culturing *P. gingivalis* biofilm on Ti discs and treating with pre-filtered sterilized Est816. (**G**-**I**) Inhibiton of *kgp*,* fimA*,* and rgpA* expression of *P. gingivalis* treated with Est816 enzymatically activated (5 and 9 U mL^− 1^) and hyperthermia-inactivated Est816 (9 U mL^− 1^) (compared with non-treatment group, *p < 0.05, **p < 0.01, ***p < 0.001, error bars in each panel represent the SD of triplicates)

Biofilm biomass was quantified using a crystal violet (CV) staining assay. *P. gingivalis* cultures, grown overnight in sTSB were adjusted to 0.5 McFarland standard. Then, 0.5 mL of the bacterial suspension was added to a 12-well cell culture plate containing sterilized Ti discs (Fig. 2F). The enzymatically active Est816 was introduced at final concentrations of 5 and 9 U mL^− 1^. Parallel wells received hyperthermia-inactivated Est816 at the same concentrations. The non-treatment group received an equal volume of sterile phosphate-buffered saline (PBS) solution. The ratio of treatment to bacterial suspension was 1:1. After 48 h of incubation in an anaerobic environment at 37 °C, free-floating cells were removed, and the biofilms were washed three times with sterile PBS. The biofilms were then fixed with 99% (v/v) methanol. The Ti discs were stained with 1% (w/v) crystal violet at room temperature for 15 min. Following staining, the biofilms were washed with PBS three times and allowed to dry for 2 h until complete evaporation of moisture. Photographic documentation of the specimens was recorded. The crystal violet was then solubilized using 95% (v/v) ethanol, and the solution was homogenized prior to absorbance measurement at 590 nm using a microplate reader. The reported values represent the mean of three independent measurements.

### Scanning electron microscopy (SEM) observation

Structured colonies within the biofilms were observed using scanning electron microscopy (SEM). The cultivation and treatment procedures followed the methods detailed above. Biofilms on the Ti discs were rinsed with PBS and fixed in 2.5% glutaraldehyde overnight. Subsequently, the samples were serially dehydrated in ethanol for a total of 75 min at increasing concentrations (30%, 50%, 70%, 90%, 100%). The dehydrated samples were then sputter-coated and examined using a field emission scanning electron microscope (S-4800, Hitachi, Japan). SEM images of the biofilm formation on the Ti discs were captured at magnifications of 1000x and 5000x. The anti-biofilm activity of Est816 was evaluated by comparing these images with those from the non-treatment group. Observations were made on three biofilms and three fields within each group.

### Fluorescence microscopy imaging

The effect of Est816 on biofilm separation was evaluated using a confocal laser scanning microscope (CLSM). A mixture solution of SYTO 9 (3.34 mM) and PI (20 mM) in a 1:1 proportion was diluted 150-fold in sterile PBS (pH 7.4) to the final working concentrations of 11 µM for SYTO 9 and 66 µM for PI. After 48 h of adhesion and biofilm formation on the glass slips, samples were covered with Live-dead staining dye and incubated for 15 min at room temperature. Images were obtained and recorded using a confocal microscope (FluoView 1000, Olympus, Japan). The Image-pro Plus computer program (Mediacybernetics, USA) was used to calculate fluorescence regions and analyze the structural organization of the microbial communities by quantifying three-dimensional biofilm image stacks. Tests were performed on three samples for each situation.

### Colony forming unit counting assay

The colony forming units of *P. gingivalis* cells on the Ti discs were evaluated by CFU assay. Three successive dilutions (1:10^3^, 1:10^4^, and 1:10^5^) were prepared. Then, 50 µL of the bacteria suspension from each dilution were spatulated on blood agar plates. Incubation proceeded at 37℃ under anaerobic conditions for 5 days. After this period, the colony-forming units (CFU mL^− 1^) were counted. Tests were performed on three discs for each situation.

### EPS measurement assay

The phenol-sulfuric acid assay was employed to quantify the concentration of glycopolymers and their conjugates. Fresh solutions of 6% (w/v) phenol in double-distilled water (ddH_2_O) and concentrated sulfuric acid were prepared. A 50 µL aliquot of *P. gingivalis* suspension, adjusted to 0.5 McFarland standard, was added to a 96-well plate. This was followed by the addition of 50 µL of enzymatically activated (5 and 9 U mL^− 1^) and hyperthermia-inactivated Est816 (9 U mL^− 1^). The cultures were incubated for 48 h. Post-incubation, the resulting biofilms were washed three times with PBS and allowed to dry for 20 min at room temperature. Subsequently, 40 µL of ddH_2_O, 40 µL of 6% phenol, and 200 µL of concentrated sulfuric acid (95-97%) were added to each well. The plate was covered with a lid, mixed by shaking at 900 rpm for 5 min at room temperature, and then incubated for 30 min to allow the color development. The resulting solution was transferred to a new 96-well plate, and the optical density was measured at 490 nm. Reported values represented the mean of three independent measurements.

### Virulence genes expression

RT-qPCR assay was used to verify the effects of Est816 on the expression of virulence genes in *P. gingivalis*, including gingipain K (*kgp*), fimbriae A (*fimA*), and gingipain R (*rgpA*). RNA was extracted from *P. gingivalis* pre-treated with different concentrations of Est816 enzymatically activated (5 and 9 U mL^− 1^) and hyperthermia-inactivated Est816 (9 U mL^− 1^) and synthesized into cDNA according to the manufacturer’s instructions. The sequences of the PCR primers used in this study were shown in Table 1. The reference gene used in the quantitative analysis of *P. gingivalis* mRNA expression was gyrase B (*gyrB*). The PCR reaction parameters were as follows: initial denaturation at 95 °C for 30 s, followed by 40 cycles of a melting step at 95 °C for 3 s and annealing/extension at 60 °C for 30 s. All samples were tested in triplicate (technical replicates of RT-qPCR on the same RNA extract).

**Table 1:** Polymerase chain reaction primers of *P. gingivalis*

Primer	Forward (5’- 3’)	Reverse (5’- 3’)
*gyrB*	AGTGGGCGTTTCTTGTGTGAA	CAGCTGAACTCCTGCATATGGA
*kgp*	ACACCTGTTGTTCGCGTGAA	AGAGGGTTGATGTGGCATGAG
*fimA*	ATAATGGAGAACAGCAGGAA	TCTTGCCAACCAGTTCCATTGC
*rgpA*	TCTTTGGCGGTTTCAGACACT	GGAGGGTGCAATCAGGACAT

### Cell culture

PDLSCs (gifts from the Central Research Laboratories of Faculty of Dentistry of The University of Hong Kong) were cultured in DMEM medium supplemented with 100 µg mL^− 1^ of the antimicrobial agent Primocin (InvivoGen, USA) at 37 °C in a 5% CO_2_ atmosphere. The culture medium was refreshed every 72 h, and cells from passages 1 to 2 were utilized for all experiments. Human oral keratinocytes (HOK) (gifts from the Central Research Laboratories of Faculty of Dentistry of The University of Hong Kong) were cultured in high glucose DMEM medium under the same conditions (37 °C, 5% CO_2_). The culture medium was also changed every 72 h, with passages 1 to 3 being used for all experiments.

### Fluorescence staining

To evaluate the cytotoxicity of Est816, HOK cells were seeded in 6-well plates at 1 × 106 cells per well and cultured for 24 h to cell adherence. The medium was then removed, and the plates were supplemented with culture medium followed by Est816 at a ratio of 1:1, in which the final working concentrations of Est816 reached to 9 U mL^− 1^. For the non-treatment group, medium and PBS were added at the same proportions. After 1, 3 and 5 days of incubation, fibrillar actin (F-actin) structures were detected using standard TRITC-phalloidin staining (Sigma, St. Louis, MO, USA). Subsequently, cells were examined under a fluorescence microscope (DM300, Leica, Germany).

### Enzyme-linked immunosorbent assay

**Fig. 3:**
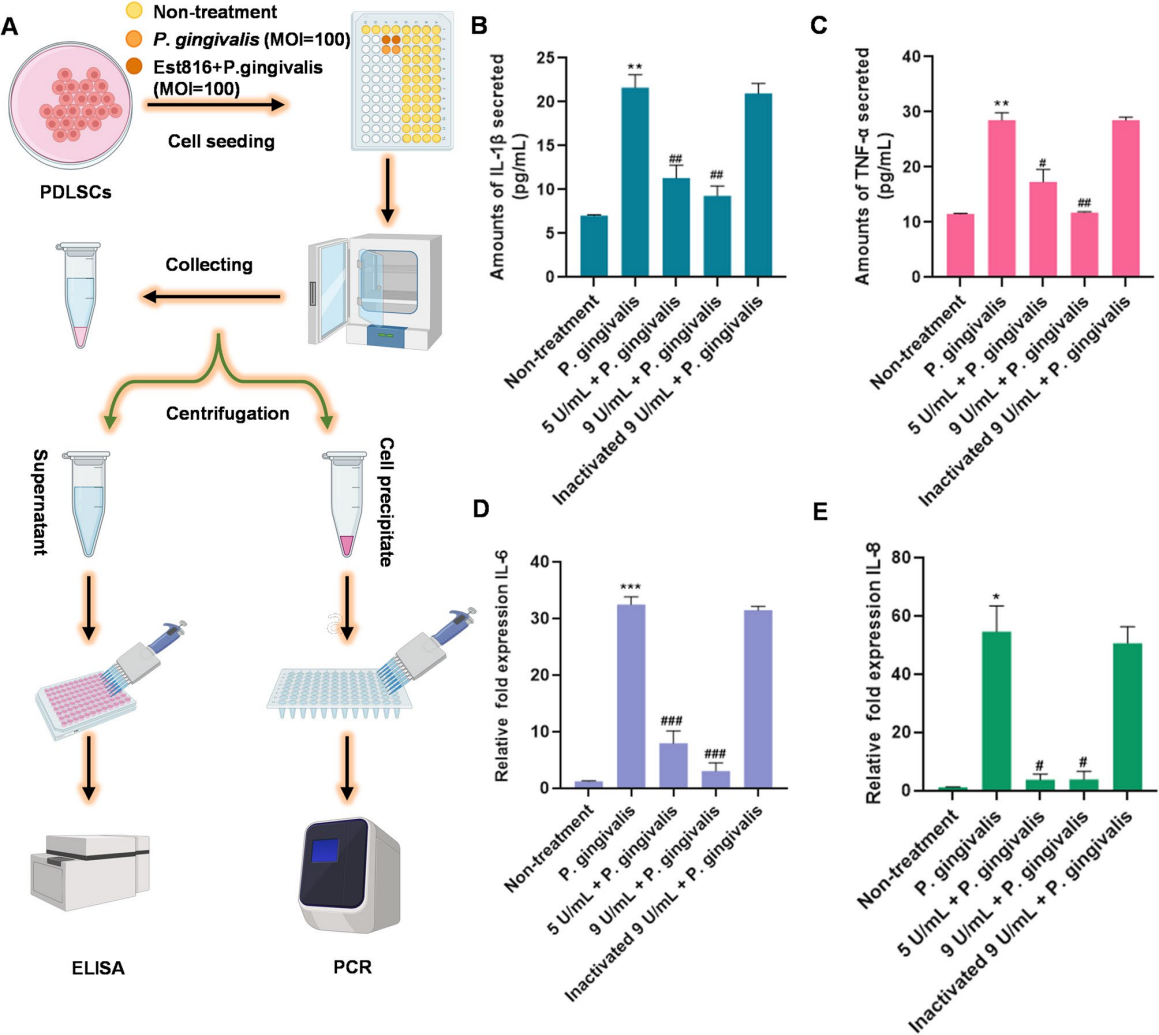
(**A**) Procedure diagram of PDLSCs culturing with *P. gingivalis* treated with or without Est816, and sample collection for ELISA and PCR assays. (**B**-**C**) Protein expression of IL-1β and TNF-𝛼 in the supernatant of PDLSCs. (**D**-**E**) Expression of IL-6 and IL-8 gene of PDLSCs under different treatments (compared with non-treatment group, *p < 0.05, **p < 0.01, ***p < 0.001; compared with *P. gingivalis* group, ^#^*p* < 0.05, ^##^*p* < 0.01, ^###^*p* < 0.001, error bars in each panel represent the SD of triplicates)

An ELISA assay was employed to determine the protein levels of interleukin-1β (IL-1β) and tumor necrosis factor-α (TNF-α) in the supernatants of PDLSCs (Fig. 3A). The experimental groups included the *P. gingivalis* group, the Est816 + *P. gingivalis* groups, and a non-treatment group. PDLSCs were seeded at a density of 5 × 10^5^ cells per well and allowed to adhere for 12 h. Subsequently, cells were co-cultured under the following conditions: with *P. gingivalis* cells at a multiplicity of infection (MOI) of 100 in the *P. gingivalis* group; with *P. gingivalis* cells (MOI of 100) and Est816 at final concentrations of enzymatically activated (5 and 9 U mL^− 1^) and hyperthermia-inactivated (9 U mL^− 1^) in the Est816 + *P. gingivalis* groups; and with sTSB medium mixed with PBS solution in the non-treatment group. Supernatants from the cell cultures were collected 12 h post-treatment. Samples were centrifuged at 15,000 g for 15 min at 4 °C to obtain clear supernatants. The concentrations of IL-1β and TNF-α in the supernatants were measured using spectrophotometry with a microplate reader at 450 nm, with wavelength correction at 540 nm. Data were normalized to the total protein concentration using an ELISA kit, following the manufacturer’s standard procedures.

### RT-qPCR

The expression levels of interleukin-6 (IL-6) and interleukin-8 (IL-8) in stimulated periodontal ligament stem cells (PDLSCs) were quantified using a quantitative reverse transcription polymerase chain reaction (RT-qPCR) assay. The treatment protocols for the three experimental groups have been previously described. Total RNA was extracted using an RNA Isolation Kit, and complementary DNA (cDNA) was synthesized utilizing the QuantiTect Reverse Transcription Kit (Qiagen, Germany). The qPCR assays were conducted on an ABI 7500 Real-Time PCR System with the QuantiNova SYBR Green PCR Kit (Qiagen, Germany). Gene expression levels were normalized to β-actin using the comparative threshold cycle (ΔΔCt) method. The primer sequences used in the study were detailed in Table 2.

**Table 2:** Polymerase chain reaction primers of PDLSCs

Primer	Forward (5’- 3’)	Reverse (5’- 3’)
ACTB	CATGTACGTTGCTATCCAGGC	CTCCTTAATGTCACGCACGAT
IL-6	ACTCACCTCTTCAGAACGAATTG	CCATCTTTGGAAGGTTCAGGTTG
IL-8	GACATACTCCAAACCTTTCCACC	AACTTCTCCACAACCCTCTGC

### Alizarin red staining

The effect of Est816 on osteoblast differentiation was examined. PDLSCs were cultured in the DMEM medium supplemented with 10% FBS (37 °C and 5% CO_2_). To induce the osteoblast differentiation, dexamethasone (10^− 7^ M), β‑glycerophosphate (10 mM) and vitamin C (50 µg mL^− 1^) were added to the medium [11]. AHL (*N*-(3-oxooctanoyl) homoserine lactone, OC8-HSL) was dissolved with dimethyl sulfoxide (DMSO). In the AHL + Est816 group, cells were treated with 50 µL AHL (50 µM) with 50 µL Est816 (9 U mL^− 1^). In the Est816 group, cells were treated with 50 µL Est816 (9 U mL^− 1^) and 50 µL DMSO. In the AHL group, cells were treated with 50 µL AHL (50 µM) and 50 µL PBS. In the non-treatment group, cells were treated with 50 µL DMSO and 50 µL PBS. PDLSCs were grown on 6-well plates at 1 × 106 cells per well. The AHL/Est816-containing medium was changed every 3 days. After 7, 14, and 28 days of culturing, cells were stained with 0.1% Alizarin Red S (Sigma, USA) at pH 6.3 for 30 min in the dark. Then, the staining solution was removed and the cell monolayer was washed twice with ddH_2_O. The staining was analyzed under a transmitted light microscope and pictures were taken with a microscope camera (Leica, Germany). For quantification of staining intensity, the cetylpyridinium chloride was added into each well and incubated for 1 h. The supernatant was transferred into a 96-well plate and optical density values for each well was detected at 570 nm by a spectrophotometer.

### Statistical analysis

All experiments were repeated at least three times. The data were presented as mean ± standard deviations (SD). Statistical analyses were performed using GraphPad Prism 8 (GraphPad Software, Inc., San Diego, USA). Comparisons among multiple groups were conducted using one-way analysis of variance with multiple comparisons by Tukey’s test followed by Bonferroni post hoc test. Statistical significance was assumed at *p < 0.05, **p < 0.01, ***p < 0.001.

## Results

### Biofilm formation of *P. gingivalis*

**Fig. 4:**
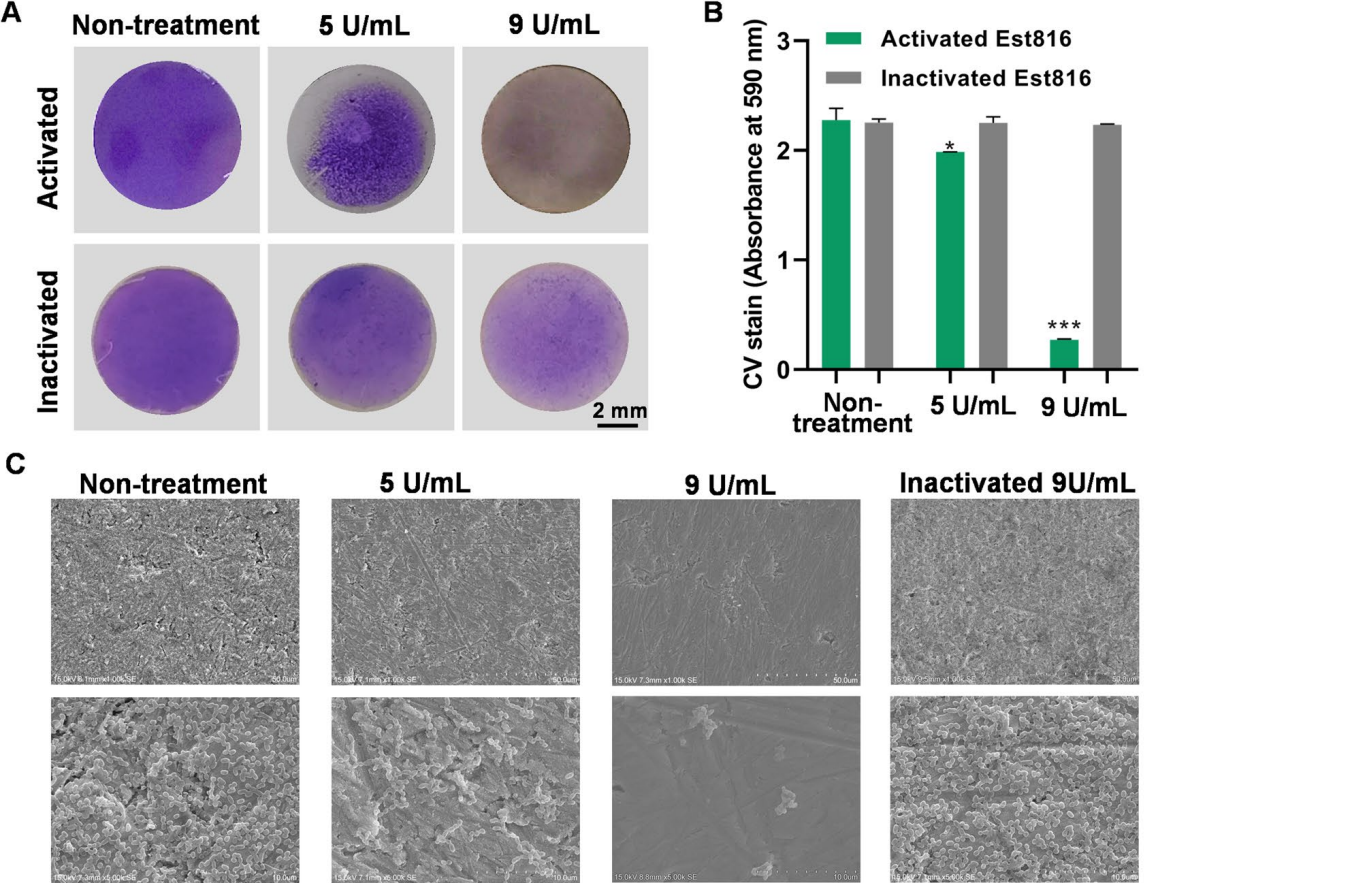
(**A**) Photographic images of crystal-violet staining of *P. gingivalis* biofilm treated with enzymatically activated and hyperthermia-inactivated Est816 on the surface of Ti discs. (**B**) Spectrophotometer quantitative analysis of crystal-violet staining of *P. gingivalis* biofilm quantity (compared with non-treatment group, *p < 0.05, **p < 0.01, ***p < 0.001, error bars in each panel represent the SD of triplicates). (**C**) SEM images of *P. gingivalis* biofilm treated with enzymatically activated of (5 and 9 U mL^− 1^) and hyperthermia-inactivated Est816 (9 U mL^− 1^) on the surface of Ti discs (magnification factor of 1000 x and 5000 x)

As shown in Fig. 4A, the crystal violet assay results indicated that the biofilm on the surface of the Ti discs in the non-treatment group was fully attached and deeply stained, while the biofilms on the surface of the Est816 groups were lightly stained and comparatively dispersed. The original metal color of Ti discs could be observed in the Est816 groups, indicating that the adhesion of *P. gingivalis* cells was inhibited. Compared with the non-treatment group, Est816 significantly reduced the amount of *P. gingivalis* biofilm in a concentration-dependent manner (*p* < 0.001) (Fig. 4B). The Ti surfaces in the hyperthermia-inactivated Est816 groups were completely attached with biofilm, the thickness of which was consistent and the structure dense. Consistent with the crystal-violet staining, SEM results showed that Est816 markedly reduced the *P. gingivalis* biofilm (Fig. 4C).

The confocal laser scanning microscopy images revealed distinct differences in biofilm structure and viability. Compared with the dense, homogeneous and intact biofilm in the non-treatment group, the biofilm in the 9 U mL^− 1^ Est816 group was sparse with bacteria scattered (Fig. 2A). The proportion of live bacteria was relatively reduced with Est816 treatment (*p* < 0.05) (Fig. 2B). Images of layer-scanning were reconstructed in the image processing software (Olympus, FV10-ASW 4.2b Viewer), showing the biofilm as a turfgrass-green three-dimensional structure. Under the treatment of Est816 at 9 U mL^− 1^, the thickness of biofilm was significantly reduced (*p* < 0.05), and the colonies were significantly dispersed (Fig. 2C).

To quantitatively verify the effects of Est816 on colony distribution and reduction of the total number of viable bacteria on the surface of Ti discs, the CFU counting method was used. The total number of adherent *P. gingivalis* cells treated with Est816 was quantified and compared with the non-treatment group. The results showed that 9 U mL^− 1^ Est816 significantly reduced the number of viable bacteria, while hyperthermia-inactivated Est816 showed no effect (Fig. 2D).

### Virulence expression of *P. gingivalis*

The main virulence factors of *P. gingivalis* include EPS, gingipain (Kgp and Rgp), fimbria (Fim). As shown in Fig. 2E, Est816 notably decreased the production of EPS of *P. gingivalis* at 9 U mL^− 1^ (*p* < 0.01). At 9 U mL^− 1^, there was a prominent downregulation of the three most important virulence-related genes (*kgp*,* fimA*, and *rgpA*), while 5 U mL^− 1^ of Est816 inhibited *kgp* gene expression only (Fig. 2G-I). These results verified the suppression effect of Est816 on the expression of the important virulence factors of *P. gingivalis*.

### Cell biocompatibility

**Fig. 5:**
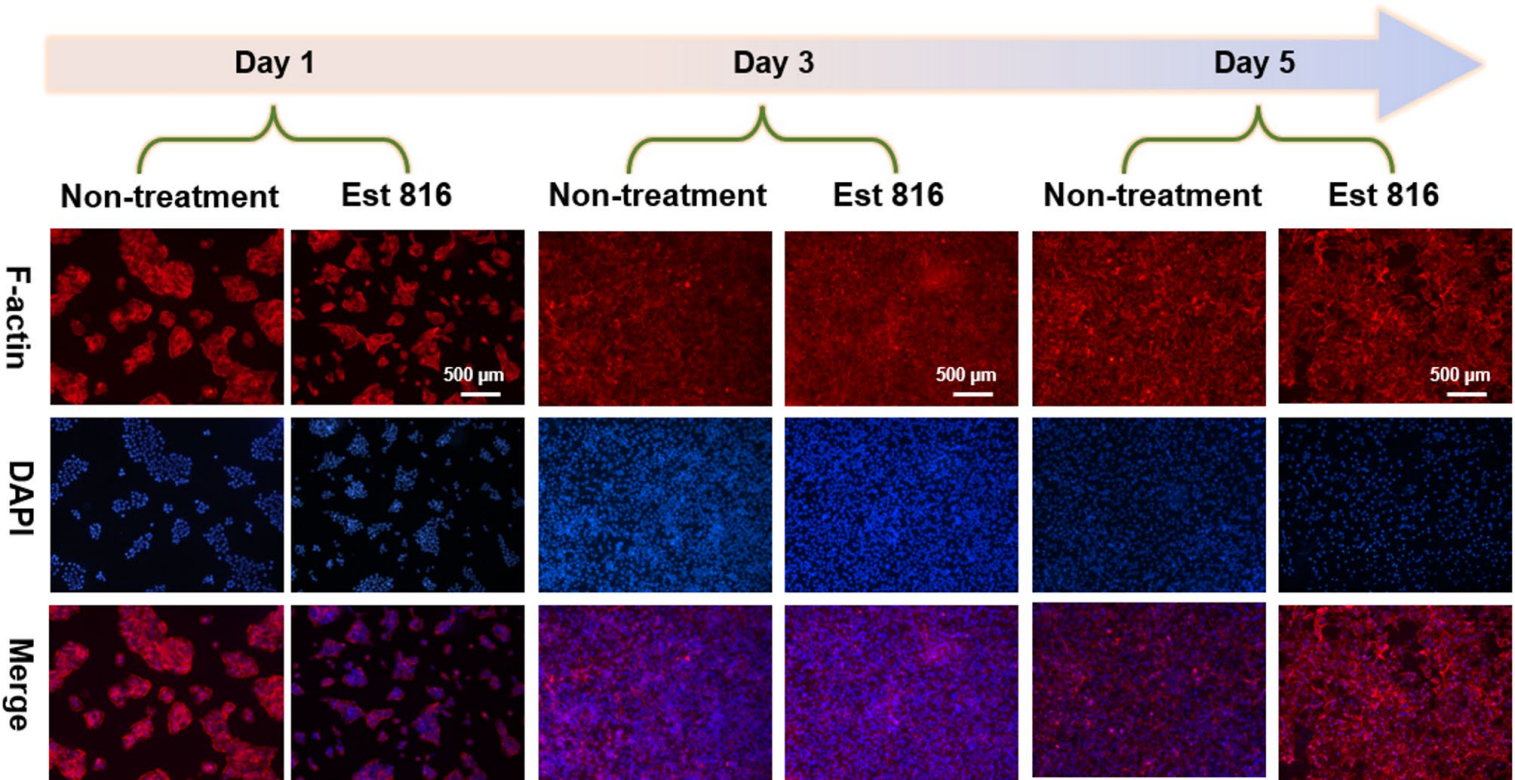
Typical appearance of human oral keratinocytes (HOKs) with blue DAPI and F-actin staining after being treated with Est816 at a concentration of 9 U mL^− 1^ for 5 days

Biocompatibility of anti-biofilm chemotherapy is critical for a successful clinical outcome. Considering that Est816 at 9 U mL^− 1^ showed excellent anti-biofilm and anti-virulence effects, the concentration of Est816 at 9 U mL^− 1^ was employed in this study. We tested the effect of 9 U mL^− 1^ Est816 on the morphology on HOK cells. As was shown in Fig. 5, compared with the non-treatment group, 9 U mL^− 1^ Est816 exerted slight effect on cell proliferation after co-incubation with HOK cells for 1, 3, and 5 days. In addition, no changes in DAPI fluorescence intensity were detected in the Est816 group compared with the non-treatment group. This result demonstrated that Est816 at a concentration of 9 U mL^− 1^ did not cause morphological or nuclear damage, indicating Est816 had a good biocompatibility.

### Immune factors expression

ELISA results showed that IL-1β and TNF-α levels in the cell supernatant of 5 and 9 U mL^− 1^ Est816 groups were significantly lower than those in the *P. gingivalis* group (*p* < 0.05). There was no significant difference in the expression of IL-1β and TNF-α between the Est816 groups and the non-treatment group (*p* > 0.05). The expression of these two inflammatory factors in the *P. gingivalis* group was higher than that in the non-treatment group (*p* < 0.01) (Fig. 3B and C). The RNA expression of IL-6 and IL-8 in PDLSCs detected by real-time quantitative PCR (Fig. 3D and E). After PDLSCs were stimulated by *P. gingivalis* cells for 12 h, the expression of IL-6 and IL-8 was significantly increased compared with the non-treatment group (*p* < 0.05). In Est816 groups (5 and 9 U mL^− 1^), the expression levels were decreased compared with the *P. gingivalis* group (*p* < 0.05). However, there was no significant difference in the expression of the two genes between the Est816 groups and the non-treatment group (*p* > 0.05). These results indicated that *P. gingivalis* cells significantly increased the inflammatory expression of PDLSCs, while Est816 of 9 and 15 U mL^− 1^ effectively reduced the expression levels of inflammation response in PDLSCs. To a certain extent, Est816 inhibited the inflammatory stimulation by *P. gingivalis*.

### Osteogenic differentiation of PDLSCs

**Fig. 6:**
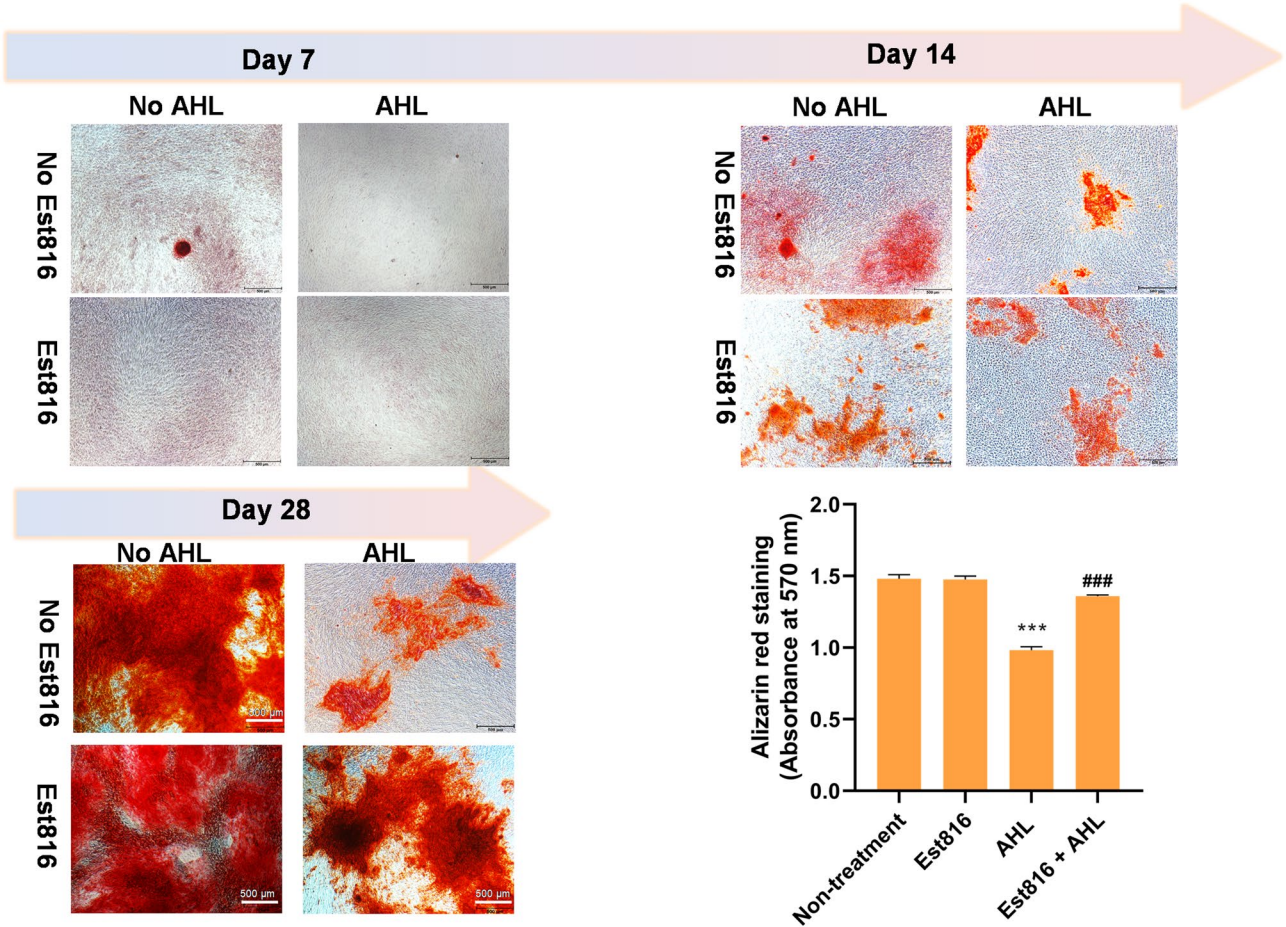
PDLSCs were cultured in osteoblast differentiation medium in the presence of AHL (OC8-HSL) with or without Est816 (9 U mL^− 1^) for the indicated time periods. For 28 days cells were fixed and stained with Alizarin red. Quantitative value was measured at 570 nm by a spectrophotometer (compared with non-treatment group, *p < 0.05, **p < 0.01, ***p < 0.001; compared with AHL group, ^#^*p* < 0.05, ^##^*p* < 0.01, ^###^*p* < 0.001, error bars in each panel represent the SD of triplicates)

After 28 days of culturing, calcium deposition appeared heavily calcified appeared dark red in both the non-treatment and Est816 group. In contrast, 50 µM AHL (OC8-HSL) suppressed the osteogenic differentiation of PDLSCs with a small amount of calcium deposition compared to the non-treatment group. Est816 exhibited no effect on osteogenic differentiation of PDLSCs on its own, but it increased the calcium deposition in the presence of AHL. Quantitative analysis confirmed the staining intensity was higher in the Est816 + AHL group compared with the AHL group, indicating that Est816 could reverse the inhibitory effect of AHL on the osteogenic differentiation of PDLSCs (Fig. 6).

## Discussion

The colonization of *P. gingivalis* and the subsequent accumulation of plaque biofilm are pivotal factors in the onset of periodontitis and peri-implantitis [12]. These biofilms, shielded by an extracellular matrix, exhibit heightened resistance to antimicrobial therapies and immune clearance, underscoring the need for strategies targeting biofilm resilience [13]. The quorum sensing system, a critical regulator of bacterial communication and virulence, plays a crucial role in biofilm development by promoting bacterial communication through signal integration [14]. Recent research into quorum sensing inhibitors, such as monosaccharides and bromofuranones, has shown promise as effective strategies for managing biofilm-related infections. These compounds display initial anti-adhesive activity, while simultaneously fostering tissue regeneration and healing at the implantation site [15, 16]. Consequently, targeting quorum sensing to disrupt the attachment, colonization, and biofilm formation of *P. gingivalis* emerges as a highly desirable approach for the prevention and treatment of periodontal diseases.

AHLs serve are essential quorum sensing molecules that promote various bacterial behaviors, including biofilm formation and virulence factor expression in gram-negative bacteria. Multiple bacterial isolates obtained from periodontal patients and oral cavity biofilms are known to produce AHLs. Muras et al. revealed that a kind of AHL- lactonase capable of degrading AHLs can inhibit the biofilm formation of periodontal pathogens and modify the microbial composition and diversity within mixed-species biofilms [5]. Our study demonstrates that Est816, a novel AHL-lactonase, exhibits strong enzymatic properties against medium- and long-chain AHLs. Notably, Est816 achieved over 94.3% degradation efficacy against the specific AHLs signal molecule produced by *P. gingivalis* [9]. Given these promising attributes, this study aims to further explore the effects of Est816 on *P. gingivalis* and its role in peri-implantitis.

Est816 significantly inhibited the aggregation and biofilm formation of *P. gingivalis* on Ti surfaces, likely by targeting and degrading AHLs. Previous studies have demonstrated that AHLs play a regulatory role in *P. gingivalis* biofilm development, particularly by coordinating bacterial adhesion [5]. The influence of AHLs and AHL-analogues on *P. gingivalis*, was also evaluated in previous works showing that when *N*-butyryl-homoserine lactone (C14-HSL) was externally added, changes in both protein expression and bacterial growth were observed [7, 17]. In this study, Est816 treatment resulted in dispersed and loosely adherent *P. gingivalis* cells on Ti discs, suggesting that AHLs hydrolysis disrupts bacterial chemical communication and blocks the initial colonization phase of biofilm formation—a critical stage for the structural integrity of mature biofilms. From a clinical perspective, this early-stage intervention offers dual advantages. First, loosely attached microbial aggregates are more susceptible to mechanical removal such as ultrasonic scaling and implant polishing, thereby enhancing the efficacy of conventional physical debridement. Second, our preliminary data indicated that combining Est816 with minocycline, metronidazole, or amoxicillin reduces the minimum inhibitory concentration of these antibiotics [10]. This aligns with findings by Zhang et al. that AHL-lactonase helped multidrug-resistant *Pseudomonas aeruginos* clinical strains change from resistant to intermediate or sensitive to antibiotics by enhancing penetration of antibiotic into biofilms [18]. These findings imply that disrupting biofilm architecture may lower the required dosage of antimicrobial agents. Thus, Est816 not only directly inhibits biofilm formation but also creates a synergistic microenvironment to potentiate existing therapeutic strategies.

Extracellular polysaccharides, gingipains and other toxins secreted by *P. gingivalis* directly damage the periodontal tissues and degrade matrix components. The expansion of the intercellular space and the enhancement of the permeability enable the invasion of bacteria and their toxic products [19]. EPS shields bacterial cells on implant surfaces, impeding antibacterial penetration and immune recognition, facilitating bacterial colonization and biofilm stability. Gingipains, including lysine gingipains (Kgp), arginine gingipains (Rgp), and fimbriae, enable *P. gingivalis* to invade periodontal tissues, degrade extracellular matrix components, and inhibit the phagocytosis of leukocyte, thereby aggravating periodontal tissues damage [20, 21]. Our study demonstrated that Est816, by degrading AHLs signaling molecules, significantly reduced *P. gingivalis* pathogenicity, thereby decreased exopolysaccharide formation and suppressed virulence factors such as gingipains (*kgp*, *rgpA*) and fimbriae (*fimA*). While the AHL-mediated QS pathway in *P. gingivalis* remains less characterized compared to the well-defined LasI/R system in *Pseudomonas aeruginosa* (*P. aeruginosa*), emerging evidence suggests the existence of a functionally analogous regulatory network in *P. gingivalis* that coordinates virulence and biofilm formation. For instance, an open reading frame with 25% identity and 48% amino acid similarity to the AHL-synthase HdtS has been identified in *P. gingivalis* W83, along with a LuxR homologue, CdhR, which regulates iron/hemin uptake through the hmu operon. Additionally, the discovery of non-classical QS synthases in other AHL-producing bacteria, such as *Psychrobacter*, suggests the potential presence of yet unidentified AHL-synthases or receptors in *P. gingivalis* that may contribute to its QS-mediated pathogenicity [7, 22]. This aligns with our findings and underscores the potential of AHL-lactonases as broad-spectrum anti-virulence agents. Notably, the anti-QS activity of Est816 mirrors that of Aii810, another AHL-lactonase previously shown to suppress virulence factors and biofilm formation in *P. aeruginosa* without affecting bacterial growth [23]. Both findings highlight the therapeutic potential of enzymatic strategies in targeting biofilm integrity and virulence, offering a promising approach to mitigate bacterial pathogenicity and tissue damage.

The interplay between AHL-mediated virulence factors and host cellular processes appears to be dynamic and triggers local immune response in peri-implant tissues [24]. IL-6 and IL-8 recruit and activate polymorphonucleocytes, leading to inflammation through the release of oxygen radicals and further damage to macrophages in periodontal tissue [25]. Increased levels TNF-α and IL-1β are proved to stimulate bone resorption process and induce osteoclast differentiation. TNF-α can induce oxidative stress in periodontal ligament stem cells to produce reactive oxygen species, which then induces mitochondrial apoptosis [26]. IL-1β has been proved to stimulate bone resorption and induce osteoclast differentiation, resulting in reduced bone levels around implants. Our study proved that Est816 mitigates *P. gingivalis*-induced inflammation in PDLSCs by downregulating pro-inflammatory cytokines (e.g., IL-6, TNF-α, IL-1β), which are known to exacerbate bone resorption and tissue degradation. This was consistent with our previous study, in which Est816 inhibited the IL-6 and TNF-α expression of human gingival fibroblasts stimulated by *A. actinomycetemcomitans*, indicating that Est816 directly reduced the inflammatory response of periodontal cells to periodontal pathogens throughout the biofilm removal phase. Moreover, Est816 targets to hydrolyze quorum sensing molecules produced by bacteria, thus showing no cytotoxic effect on HOK cells, indicating Est816 had a good biocompatibility.

Bacterial cells residing within biofilms have been demonstrated to compromise osteoblast viability, activate osteoclasts, and initiate bone resorption. This multifactorial condition poses a significant challenge to the long-term success of dental implants. Hence, we determined the osteogenic effect of Est816 on PDLSCs. Est816 showed no obvious effect on osteogenic differentiation of PDLSCs, while Est816 indirectly induced osteogenic differentiation by neutralizing AHL (OC8-HSL) that inhibit mineralization and showing a more profound calcium deposition. A previous study reported high concentration of AHLs inhibited osteoblast differentiation by promoting mitochondrial-dependent apoptosis in pre-osteoblastic MC3T3-E1 cells. The mechanical may due to AHLs elevated [Ca2+]i levels and blocked the cell-to-cell spatial autocorrelation in osteoblasts [27]. Hence, we speculated Est816 exhibited osteogenic behavior indirectly by targeting and hydrolysis AHLs in the bacterial infection microenvironment, thereby preventing excessive bone resorption.

This study had certain limitations. While *P. gingivalis* is a keystone pathogen, peri-implant biofilms are polymicrobial communities where interspecies interactions modulate virulence and resistance. Our single-species experimental design simplifies these complex dynamics, potentially underestimating the ecological role of AHLs in cross-species communication. Future work should employ multi-species biofilm models to validate Est816’s efficacy in a clinically representative microbial ecosystem. Additionally, while Est816’s enzymatic degradation of AHLs prevents osteoclast activation and bone loss, its long-term stability and activity in the dynamic oral environment warrant further investigation. This study primarily focused on the preventive potential of Est816 by targeting initial biofilm formation. However, we acknowledge that its therapeutic application against established biofilms requires systematic investigation. In clinical settings, the treatment of periodontal diseases often necessitates the disruption of mature biofilms. This is due to the known challenges related to biofilm penetration and the protective matrix of mature communities. Future studies should aim to optimize treatment parameters to enhance therapeutic efficacy, potentially leveraging successful strategies used with other quorum-quenching enzymes.

From a clinical treatment perspective, our findings demonstrate that Est816 represents a novel adjunct to conventional periodontal therapy by specifically targeting the initial bacterial adhesion phase—the most vulnerable and clinically actionable stage of biofilm development. When combined with mechanical debridement, Est816’s anti-biofilm and quorum-quenching properties could potentially enhance the efficacy by preventing rapid bacterial recolonization. Further development should focus on the sustainable strategy to enhance the durability of periodontal therapy while preserving microbial ecology.

## Conclusion

In conclusion, this study demonstrated that quorum quenching via Est816 as a multifaceted therapeutic strategy that not only disrupts *P. gingivalis* biofilm formation and virulence but also attenuates host inflammatory responses and promotes tissue repair. These dual anti-virulence and pro-regenerative properties position Est816 as a potential candidate for managing peri-implant diseases.

## Data Availability

The datasets used and/or analyzed during the current study are available from the corresponding author on reasonable request.

## Abbreviations

AHL: N-acyl homoserine lactone
P. gingivalis: Porphyromonas gingivalis
ATCC: American Type Culture Collection
Ti: Titanium
SDS-PAGE: Sodium dodecyl sulfate-polyacrylamide gel electrophoresis
DMSO: Dimethyl sulfoxide
PBS: Phosphate-buffered saline
SEM: Scanning electron microscopy
CLSM: Confocal laser scanning microscope
CFU: Colony forming unit
EPS: Extracellular polysaccharides
RT-qPCR: Quantitative reverse transcription polymerase chain reaction
PDLSCs: Human periodontal ligament stem cells
HOKs: Human oral keratinocytes
ELISA: Enzyme-linked immunosorbent assay
IL-1β: Interleukin-1β
TNF-α: Tumor necrosis factor-α
IL-6: Interleukin-6
IL-8: Interleukin-8
MOI: Multiplicity of infection
DAPI: 4’,6-diamidino-2-phenylindole
F-actin: Fibrillar actin
OC8-HSL: N-(3-oxooctanoyl) homoserine lactone
DMEM: Dulbecco’s Modified Eagle Medium
FBS: Fetal bovine serum
